# DIMet: an open-source tool for differential analysis of targeted isotope-labeled metabolomics data

**DOI:** 10.1093/bioinformatics/btae282

**Published:** 2024-04-24

**Authors:** Johanna Galvis, Joris Guyon, Benjamin Dartigues, Helge Hecht, Björn Grüning, Florian Specque, Hayssam Soueidan, Slim Karkar, Thomas Daubon, Macha Nikolski

**Affiliations:** University of Bordeaux, CNRS, IBGC UMR 5095, Bordeaux, France; University of Bordeaux, Bordeaux Bioinformatics Center CBiB, Bordeaux, France; University of Bordeaux, INSERM, BPH U1219, Bordeaux, France; Medical Pharmacology Department, Bordeaux University Hospital, Bordeaux, France; University of Bordeaux, Bordeaux Bioinformatics Center CBiB, Bordeaux, France; RECETOX, Faculty of Science, Masaryk University, Brno, Czech Republic; Galaxy Europe, University of Freiburg, Freiburg, Baden-Württemberg, Germany; Galaxy Europe, University of Freiburg, Freiburg, Baden-Württemberg, Germany; Bioinformatics Group, Department of Computer Science, Albert-Ludwigs-University Freiburg, 79110 Freiburg, Germany; University of Bordeaux, CNRS, IBGC UMR 5095, Bordeaux, France; University of Bordeaux, Bordeaux Bioinformatics Center CBiB, Bordeaux, France; University of Bordeaux, CNRS, IBGC UMR 5095, Bordeaux, France; University of Bordeaux, Bordeaux Bioinformatics Center CBiB, Bordeaux, France; University of Bordeaux, CNRS, IBGC UMR 5095, Bordeaux, France; University of Bordeaux, CNRS, IBGC UMR 5095, Bordeaux, France; University of Bordeaux, Bordeaux Bioinformatics Center CBiB, Bordeaux, France

## Abstract

**Motivation:**

Many diseases, such as cancer, are characterized by an alteration of cellular metabolism allowing cells to adapt to changes in the microenvironment. Stable isotope-resolved metabolomics (SIRM) and downstream data analyses are widely used techniques for unraveling cells’ metabolic activity to understand the altered functioning of metabolic pathways in the diseased state. While a number of bioinformatic solutions exist for the differential analysis of SIRM data, there is currently no available resource providing a comprehensive toolbox.

**Results:**

In this work, we present DIMet, a one-stop comprehensive tool for differential analysis of targeted tracer data. DIMet accepts metabolite total abundances, isotopologue contributions, and isotopic mean enrichment, and supports differential comparison (pairwise and multi-group), time-series analyses, and labeling profile comparison. Moreover, it integrates transcriptomics and targeted metabolomics data through network-based metabolograms. We illustrate the use of DIMet in real SIRM datasets obtained from Glioblastoma P3 cell-line samples. DIMet is open-source, and is readily available for routine downstream analysis of isotope-labeled targeted metabolomics data, as it can be used both in the command line interface or as a complete toolkit in the public Galaxy Europe and Workfow4Metabolomics web platforms.

**Availability and implementation:**

DIMet is freely available at https://github.com/cbib/DIMet, and through https://usegalaxy.eu and https://workflow4metabolomics.usegalaxy.fr. All the datasets are available at Zenodo https://zenodo.org/records/10925786.

## 1 Introduction

Stable isotope-resolved metabolomics (SIRM) has strongly contributed in recent years to advance our understanding of metabolic regulation in metabolism-related pathologies such as cancer (Méndez-Lucas *et al.* 2020), diabetes, or cardiovascular diseases (Balcells *et al.* 2019). Closely related to conventional metabolomics, SIRM uses an isotope-labeled substrate to track isotope-labeled metabolic substrates through downstream pathways (Lorkiewicz *et al.* 2019) and often concerns targeted quantification of a subset of known compounds (Shi *et al.* 2020). Targeted SIRM, more sensitive to signals close to the detection threshold, is often used to examine the metabolic effects of a pathological state or of an induced biological change (Giacomoni *et al.* 2015, Jang *et al.* 2018).

In SIRM experiments, either cells (*in vitro*) or the organism (*in vivo*), are fed with a ^13^C (or other stable isotope) labeled substrate and quantification is typically achieved using liquid chromatography–mass spectrometry and more rarely, nuclear magnetic resonance or gas chromatography–mass spectrometry (Krämer *et al.* 2018). In terms of data, not only the total metabolite abundances, but also the quantified incorporation of the ^13^C isotope label, are acquired in SIRM experiments. This dual information allows to study differences both in terms of total metabolite abundances and of the integration speed of the labeled carbons between conditions of interest, allowing to uncover biomarkers and understand metabolic changes associated with a particular condition or in time. Indeed, differences in isotope enrichment, changes in the labeling patterns, or differences in the contribution of nutrients to a metabolite pool, provide crucial knowledge of the cell’s metabolic activity and state (Buescher *et al.* 2015, Bruntz *et al.* 2017).

An isotopologue is a unique variant of the metabolite with a specific number of stable isotopes and mass, function of the number of labeled carbon atoms. Distributions of stable isotopes for a given metabolite, which defines labeling patterns, and commonly represented as mass distribution vectors (MDVs), represent the fractional abundances of isotopologues for each mass-to-charge (m/z) ratio and normalized to the sum of all possible isotopologues. Thus, the labeling pattern of a metabolite with n carbon atoms is $\left(M_{0}{,\ldots ,M}_{n}\right)$ with $M_{i}$ being the relative abundances of the corresponding isotopologues ranging from no labeled carbons to all labeled carbons, i.e. ^13^C. At the isotopic steady state MDVs become time-invariant and their values can be considered as proxy of metabolic fluxes and structure of the metabolic network. Time-course experiments during the isotopic nonsteady state provide information on changes in patterns of labeling, the directionality of reactions, and incorporation speed before reaching the steady state.

Data correction to ensure accurate quantification of isotopic patterns and to minimize potential biases such as natural isotope abundances and overlap of mass spectra, is performed using software tools such as e.g. IsoCor (Millard *et al.* 2019) or PolyMID (Jeong *et al.* 2021), and results in MDVs where the labeling pattern is attributable only to the tracer.

Downstream bioinformatics analyses take such corrected MDVs as input and aim to shed light on substrate contributions, pathway’s activity up to SIRM-based metabolic flux analysis (Lorkiewicz *et al.* 2019). While metabolic fluxes can be calculated using dedicated mathematical models with stoichiometric constraints, such analyses are computationally intensive (Millard *et al.* 2020, Lugar and Sriram 2022); and the complexity of building and parameterizing the most plausible model based on kinetic equations should not be underestimated (Yuan *et al.* 2009, Millard *et al.* 2021). Alternatively, it is recognized by the scientific community that analysis of ^13^C labeling patterns is sufficient to provide information on relative pathway activities (Buescher *et al.* 2015).

Most of the available bioinformatics pipelines are dedicated to the analysis of conventional metabolomic data, such as the popular MetaboAnalyst (Pang *et al.* 2021), though some tools for tracer data analysis have been proposed. Open-source tools specifically designed for tracer metabolomics differential analysis include isoplot and univariate scripts integrated in the Workflow4Metabolomics suite (Giacomoni *et al.* 2015, Guitton *et al.* 2017), DynaMet (Kiefer *et al.* 2015), and TraVis Pies (De Craemer *et al.* 2022). Unfortunately, options available for statistical significance analysis in these tools are mainly parametric tests, which are inadequate for data where one cannot assume a Gaussian distribution and sample size is often small. Some even completely lack statistical support (DynaMet) or do not support correction for multiple testing (TraVis Pies). Proprietary software tools for isotope-labeled data analysis are also available such as PollyPhi^TM^ (Agrawal *et al.* 2019).

Importantly, existing methods often do not account for the specificities of isotopologue-based analysis, e.g. time-course analysis for dynamic tracer experiments is not covered. Thus, there is a strong need in the community for a one-stop comprehensive resource for targeted tracer metabolomics data analysis. To fill this gap, we have developed DIMet, a bioinformatics tool for differential analysis of isotopically resolved metabolomics data.

**Figure 1.**
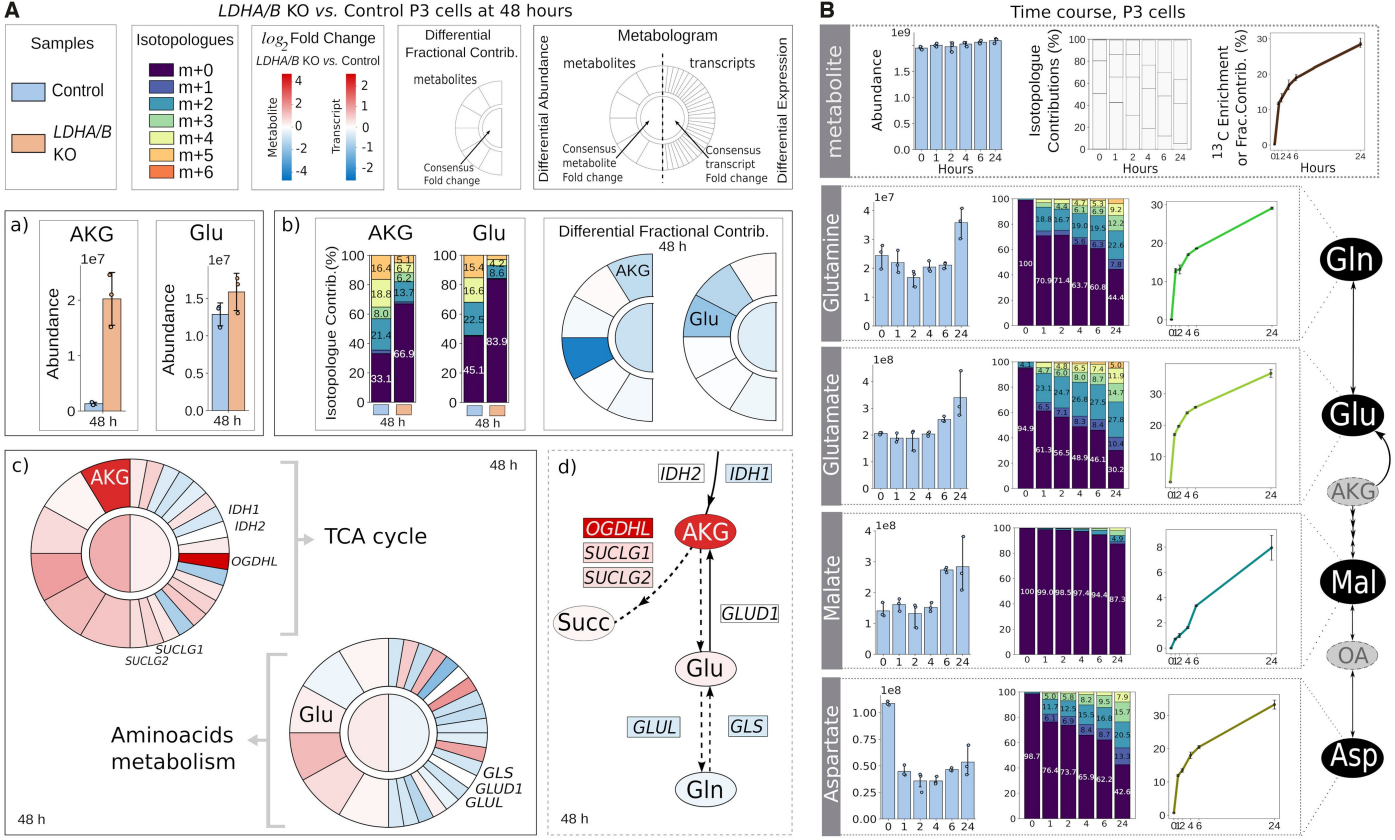
(A) Comparison between LDHA/B KO and control samples at 48 h. Control and LDHA/B KO samples' legend is in the upper-left corner of panel A ('Samples'). The m+0 is the unlabeled isotopologue. Isotopologue scale is represented in the 'Isotopologues' upper section of panel A. The total abundance is represented with comparative bars (a). Isotopologue contributions are shown as stacked bars, these and the differences in fractional contributions are interpreted jointly (b). Highlighted DAMs, AKG, and Glu, are shown within pathway specific metabolograms (c). The extracted sub-network shows DAMs as ovals, DEGs (Differentially Expressed Genes) as rectangles, and dotted arrows as hypothesized slow fluxes (d), see text. (B) Time-course analysis of P3 cells. The three types of measures are shown across time, by metabolite. The isotopologue contributions have the same color key as Isotopologues legend in 1.A. The DAMs, exhibiting a significant difference in at least one $t_{x+1}\;$versus $t_{x}$ comparison, are shown, alongside a partially reconstructed metabolic map (right). No transcriptome was available for this experiment. AKG, alpha-ketoglutarate; Succ, succinate; Glu, Glutamate; Gln, Glutamine; OA, Oxaloacetate; Mal, Malate; Asp, Aspartate.

DIMet is designed to perform differential and time-series analyses of corrected isotopic labeling data obtained from targeted experiments. It uses a rigorous statistical framework to identify metabolites that are differentially labeled between conditions. Notice that DIMet does not aim to perform flux analyses. The package can handle univariate, bi-variate and multivariate data, allowing for the analysis of individual metabolites or entire metabolic pathways. The workflow of DIMet includes several visualization tools and statistical analyses suitable for tracer data to determine the significance of abundance or labeling differences to obtain Differentially Abundant—or labelled—Metabolites (DAM). Finally, DIMet provides integration with transcriptomic data through the metabolograms (Hakimi *et al.* 2016) (see Fig. 1).

## 2 Pipeline description and implementation

### 2.1 DIMet workflow

The architecture of DIMet is shown in Supplementary Fig. S1 and it accepts three types of data as input:

1. corrected isotopologues, that can be provided either as absolute values $\left(M_{0},...,M_{n}\right)$ or as proportions (isotopologue contributions) $c_{i}=\frac{M_{i}}{\sum_{j=0}^{n}M_{j}\;}\;$ provided for each $M_{i}$;
2. total metabolite abundances defined as $m_{i}=\sum_{j=0}^{n}M_{j}\;$ for each measured metabolite;
3. fractional contributions defined for each measured metabolite as $\varphi_{i}=\frac{\sum_{j=0}^{n}{(c}_{j}\;*\;j)\;}{n}$.

These inputs are provided as tab-delimited files accompanied by a configuration file that specifies the analyses parameters. If additional data preprocessing is needed, such as computing total abundances or converting the input format to accommodate DIMet requirements, an accompanying preprocessing tool TraceGroomer is also provided and linked from the main GitHub repository of DIMet.

A typical study starts with exploratory analyses of (i) the total metabolite abundances and (ii) the labeling speed. For the former, comparisons of $m_{i}$ values between samples from different conditions using barplots are proposed. For the latter,^13^C enrichment and isotopologue contribution plots are generated from $c_{i}$ vectors and $\varphi_{i}$ values. These two outputs should be interpreted jointly in order to gain the understanding of the speed of the labeled substrate incorporation: an increase in fractional contribution is indicative of a relatively faster labeling speed, and intuitively, faster labeling means higher flux (Jang *et al.* 2018).

Such data exploration is followed by rigorous statistical analyses for which DIMet offers both univariate and multivariate statistics. Statistical analysis can be performed for pairs of samples both for data from two different conditions as well as for all pairwise consecutive time-points $t_{i+1}$ versus $t_{i}$ from a time-course acquisition. Statistical significance of differential abundances can be computed using either parametric or nonparametric tests (univariate statistics): *t*-test, Kruskal–Wallis, Mann–Whitney, Wilcoxon’s signed rank test, Wilcoxon’s rank sum test and permutation test are currently offered (see the Supplementary Material). Resulting *P*-values can be adjusted for multiple comparisons using Benjamini–Hochberg (Benjamini and Hochberg 1995) or any other FDR method available in the statsmodels library (Seabold and Perktold 2010). Multi-group analysis allows to directly compare three or more conditions. DIMet implements this by applying the Kruskal–Wallis test, a nonparametric alternative to ANOVA: indeed, the assumptions of data normality and homoscedasticity are seldom fulfilled in tracer metabolomics datasets, and the sample size is often small. The bivariate analysis allows to compare the entire MDV profiles using the Spearman correlation test. A multivariate analysis is also proposed by producing a Principal Components Analysis (PCA) graph, applicable to total metabolite abundances and fractional contributions.

### 2.2 Implementation

DIMet has been developed in Python 3.9.7 and 3.10. It is available both as a stand-alone package and a suite of Galaxy tools. The stand-alone version of DIMet can be downloaded as a PyPI or as a Conda package; alternatively, it can be used via Docker or Singularity containers. The stand-alone version has been tested in Ubuntu 22.04 and Mac OS Ventura 13.5.1. A user-friendly Galaxy version is accessible at https://usegalaxy.eu, and in the section *Isotopic Studies* at https://workflow4metabolomics.usegalaxy.fr.

## 3 Use case: glioblastoma metabolic adaptation under hypoxia

### 3.1 Materials

We illustrate the use of DIMet on data acquired in our previous work (Guyon *et al.* 2022) to study glioblastoma patient-derived stem-like cells (P3). Glioblastoma (GB) is a malignant brain tumor with a low survival rate despite heavy treatment. In Guyon *et al.* (2022), we were interested in explaining to what extent the central carbon metabolism is altered in tumoral cells, by selectively deleting lactate dehydrogenases, thus blocking fermentation. Here we present how the corresponding SIRM data analyses can be performed in DIMet, both for comparisons between conditions and for time-course experiments.

1. First, the role of lactate dehydrogenases (*LDHA, LDHB*) was investigated using ^13^C6-glucose as substrate in P3 control and double *LDHA/B* KO cells under hypoxia. Both SIRM and transcriptome RNAseq datasets were obtained in triplicates at 48 h.
2. Second, for the P3 wild type cell line a time-course experiment was performed. Cultures were fed with ^13^C6-glucose and the SIRM data was acquired at 0, 1, 2, 4, 6, and 24 h.

In both cases generated data consisted of total abundances, fractional and isotopologues’ contributions.

### 3.2 Step-by-step analyses and results

Both datasets were processed by DIMet: dataset (1) for the comparison of control and LDHA/B conditions and dataset (2) to study the labeling speed using a time-series setup. Jointly, this allowed an integrated overview of TCA (tricarboxylic acids) cycle and amino acids, improving the biological interpretation of the role of lactate dehydrogenases in metabolic rewiring of glioblastoma. For the purpose of this use case, we highlight here the analyses steps available in DIMet and the results they can yield. All the presented analyses are fully reproducible following the steps detailed on the DIMet Wiki page of the GitHub repository.

#### 3.2.1 Differential analyses

Here we illustrate the comparison between *LDHA/B* KO and Control P3 tracer metabolomics data at 48 h and their integration with the corresponding transcriptomics datasets.

- First, we compared total metabolite abundances between *LDHA/B* KO and control P3 datasets, using the permutation test, yielding 21 statistically differentially abundant metabolites (DAMs). As shown in Fig. 1A(a), alpha-ketoglutarate (AKG) levels were significantly increased in the KO condition ($p_{\mathrm{adj}}\;\leq \;0.05$).
- Second, in order to elucidate whether this increase is due to accumulation or to overproduction, it was necessary to investigate the labeling speed. We have thus analyzed both isotopologue and fractional contributions’ datasets, using the Wilcoxon’s rank sum test and the permutation test, respectively. As shown in Fig. 1A(b) we observed a significant decrease in the ^13^C enrichment for AKG and Glutamate, which was interpreted as a decline in labeling speed, suggesting, either a slower flux through their associated reactions, or an existence of an alternative path producing both metabolites from unlabeled sources.
- Finally, to disambiguate these two possibilities, network-based analyses was performed using metabolograms (to enable this analysis, RNAseq data for the same samples was processed to obtain the set of differentially expressed genes) for TCA cycle and amino acids metabolism, see Fig. 1A(c). Network projection of these results indicated that a significant dysregulation of key genes encoding for the enzymes downstream and upstream of AKG (i.e. *OGDHL, GLUL*) favored the hypothesis of slow flux from AKG, which accumulates in the cell. Globally, the TCA cycle and the amino acids metabolism were significantly perturbed when the *LDHA* and *LDHB* genes were deleted in P3 cells exposed to hypoxia at 48 h.

#### 3.2.2 Time-course analysis

In the time-course analysis of the control P3 cells, the consecutive timepoints were automatically compared ($t_{i+1}$ versus $t_{i})$ using the Wilcoxon’ rank sum test, and that for all three types of input data (Fig. 1B). MDV profiles underwent bivariate analysis (Spearman test). Malate total abundance, but not its labeling, increased. Interestingly, aspartate levels dropped, while its labeling increased. These findings suggest that unlabeled sources are used for synthesizing malate, whereas aspartate is consumed with time. Moreover, glutamate’s ^13^C enrichment has raised. This latter observation combined with a linear relationship between MDVs across all comparisons (e.g. 6 h versus 4 h: ${\rho =0.94,\;p}_{\mathrm{adj}}=0.006$), indicated a sustained increase in labeling. A partial reconstruction of the metabolic map (Fig. 1B, right) provided a helpful mechanistic overview of this experiment.

## 4 Conclusion

The proposed workflow offers comprehensive functionalities for the analysis of tracer metabolomics data, covering differential and time-course data analysis capabilities as well as integration with transcriptomics data. We showcased its utility on a specific use-case to investigate glioblastoma metabolic adaptation under hypoxia. The tool is easy to install and is also available under a user-friendly Galaxy interface.

## Supplementary Material

btae282_Supplementary_Data
