# Supplementary material for: DIMet: an open-source tool for differential analysis of targeted isotope-labeled metabolomics data
Source: Bioinformatics. 2024 Apr 24;40(5):btae282. doi: 10.1093/bioinformatics/btae282 (PMC11109473; doi:10.1093/bioinformatics/btae282)
Supplement: btae282_Supplementary_Data [file btae282_supplementary_data.pdf]

DIMet: An open-source tool for Differential analysis of targeted Isotope-labeled Metabolomics data
Supplementary material

J. Galvis, J. Guyon, B. Dartigues, H. Hecht, B. Grüning, F. Specque, H. Soueidan, S. Karkar, T. Daubon and M. Nikolski

Contents

S1 DIMet architecture . . . 1
S2 Expected directory structure to run DIMet from the command line . . . 2
S3 Statistical analyses and their outputs . . . 2
S3.1 Univariate analyses . . . 3
S3.2 Bi-variate analyses . . . 3
S3.3 Multiple tests correction methods . . . 5
S3.4 Output table of the DIMet univariate analyses . . . 5
S3.5 Output table of the DIMet bi-variate analyses . . . 5

The present document provides complementary information to that in the Wiki page of DIMet documentation. Its goal is to provide additional details as for (i) the global architecture of the tool, (ii) the directory and configuration file structure for the command-line version, (iii) the options related to the statistical tests proposed for different analyses, and (iv) the output of these statistical analyses.

S1 DIMet architecture

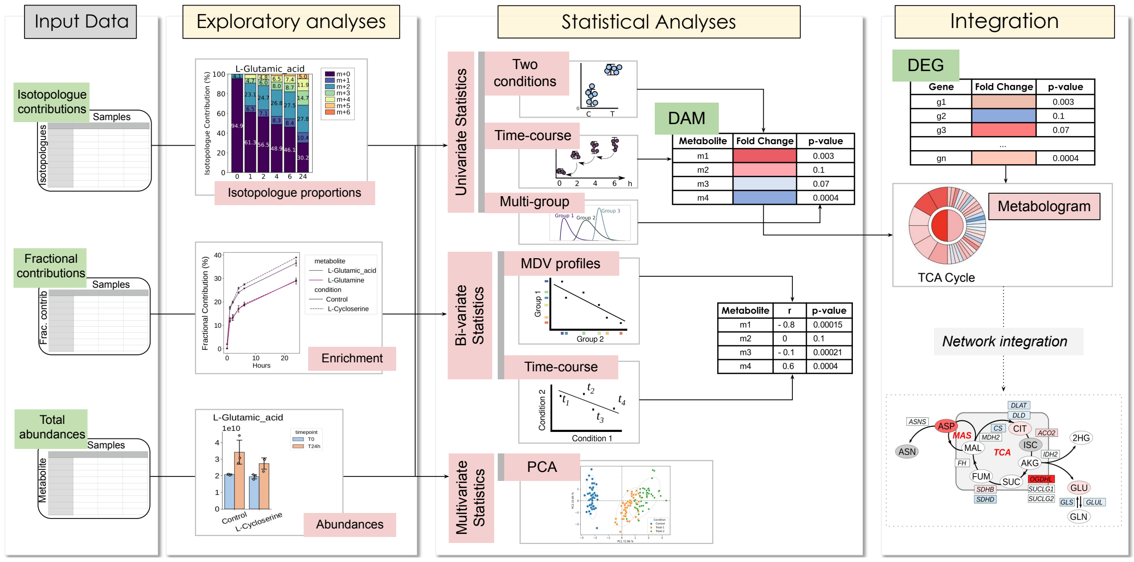

Supplementary Figure 1: DIMet offers different options for exploratory and statistical analyses (central-left and central panels, respectively) of SIRM data. Both differences between conditions as well as time-course experiments can be analysed with univariate or bi-variate statistics (central panel). Downstream integration with transcriptome through metabolograms is also available (right panel), allowing projection onto a metabolic network (by external tools).

S2 Expected directory structure to run DIMet from the command line

A specific folder structure is necessary for using DIMet in the command line. The Supplementary figure 2 details the required folder structure.

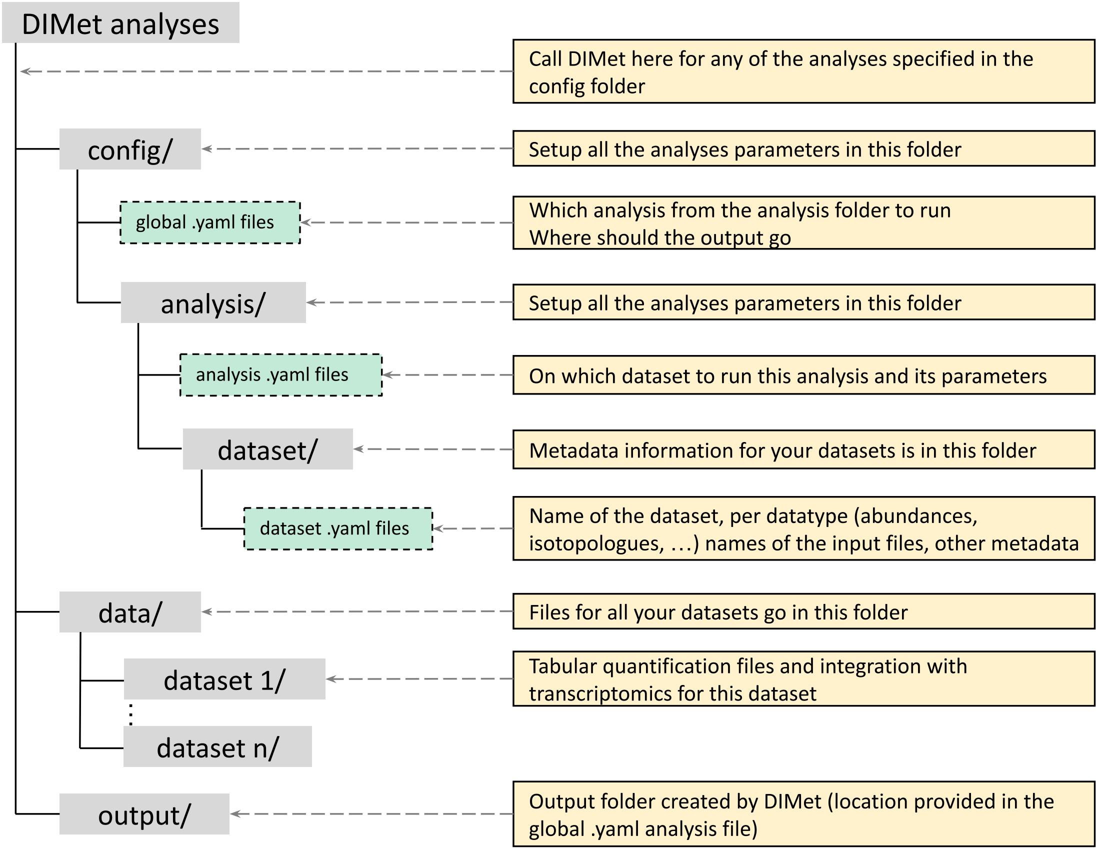

Supplementary Figure 2: Using DIMet in the command line requires to follow the predefined folder structure for your data and all the parameters that have to be indicated in the configuration files. Further details are provided in the Wiki page alongside downloadable templates.

The Supplementary figure 2 can be read as an instruction from top to bottom, detailing all the necessary files and their location. It covers the data itself, its metadata, the configuration files for the analyses to be run, etc.

Importantly, we provide **starter templates for all these files** downloadable from Zenodo to facilitate the use of DIMet. They can be easily modified according to your own data and analyses. The formats of each type of data file in the dataset subfolder are described in detail in the Wiki section data-files.

S3 Statistical analyses and their outputs

This section presents key concepts and recommendations regarding the **statistical tests for comparing groups of samples** with DIMet (Supplementary figure 1). The mathematical details, with few exceptions, are out of the scope of this document: we provide supplementary references which the reader can refer to.

S3.1 Univariate analyses

Goal of the analysis: exhibit differences between samples from 2 conditions, that is evaluate whether there is a significant increase or decrease in the abundances or the labeling of the metabolites between them.

Available univariate analyses:

a. Pairwise differential analysis: comparing 2 groups of samples

b. Time course analysis: comparing samples between consecutive time points

c. Multi-group analysis: comparing samples from more than 2 groups

General considerations for establishing the statistical significance. Targeted metabolomics data are not well suited for being analysed with parametric statistical tests[1]. Indeed, abundance values have the following characteristics: they take exclusively positive values, the distribution is not symmetrical, the variance is not homogeneous across distinct groups of values. All these elements indicate that abundance values correspond to highly skewed continuous distributions, such as e.g. the gamma distribution. Moreover, not all bell-shaped distributions are normal: for example, proportions follow a beta distribution[2].

Following these general considerations, the pairwise differential analysis and the time course analysis (a,b) share the same repertoire of tests to establish statistical significance, whereas the multi-group analysis (c) uses the Kruskal Wallis test. As a rule of thumb, we recommend considering the ranksum test as the data acquired in targeted metabolomics experiments often corresponds to its usage recommendation. It is also robust against outliers and heavy tail distributions.

The Supplementary table 1 shows the correspondences between type of analysis and statistical test. DIMet offers classical statistical tests from scipy.stats (ranksum, Wcox, MW, KW, BrMu and KW); additionally, DIMet implements disfit and prm-scipy, described in the same Supplementary table.

S3.2 Bi-variate analyses

Goal of the analysis: exhibit the correlation between samples from 2 variables, that is evaluate whether there is a significant linear relationship of the abundances or the labeling of the metabolites between them.

Available bi-variate analyses:

a. MDV[3] profile comparison between 2 conditions

b. MDV profile comparison between 2 consecutive time-points

c. Metabolite time-course profile (of total abundances and mean enrichment fractional contributions) comparison between 2 conditions

Specifics of the internal data processing for the bi-variate analysis. The following is performed for each metabolite (Supplementary table 2): In (a, b) the MDV profiles -obtained from the isotopolgue proportions- are compared between two conditions or two consecutive time-points, respectively. In (c), using the total metabolite abundances (or fractional contributions), the two sets of time-wise values between the two conditions are compared. The user can choose between the

[1]When using a Parametric test (such as t-test), assumptions of normality, homoscedasticity (homogeneity of variance) and independence must be fulfilled. A normal distribution is bell shaped AND has a mean=0 AND negative and positive values symmetrically frequent.

[2]Under certain parameters a beta distribution is bell-shaped and symmetrical with mean=0.5.

[3]Mass isotopomer Distribution Vector

Type of analysis	Test abbreviation	Full Name	Null hypothesis	Principle	Usage recommendation
Pairwise or Time-course	ranksum	Rank sum test (Wilcoxon's)	Two populations have the same distribution.	Based on the order in which the values from the two conditions fall	Unpaired samples. Equal or unequal sized samples. Any number of variables.
	Wcox	Wilcoxon's signed rank	Differences across two matched populations are close to 0.	Based on the signs and positions of the matched differences.	Paired or related -non independent- samples (check your experimental design). Any number of variables.
	MW	Mann Whitney	Two populations have the same distribution.	Based on the order in which the values from the two conditions fall.	Independent samples; a.k.a "the non-parametric version of the t-test". Assumes equal variances. Any number of variables.
	KW	Kruskal-Wallis	Two populations share the same medians.	Based on the difference in the group-wise order totals	Unpaired, independent samples. Works better with $n \geq 5$. Any number of variables.
	BrMu	Brunner-Munzel	Two populations share the same range of values.	Difference in the averaged orders of the values, normalized by the variances.	Unpaired, independent samples, being $n \geq 10$. Highly similar to the Mann-Whitney test, but does not require equal variances.
	disfit	Fitting of a distribution to the z-scores	Data follows the specified distribution.	Identify outliers using the best fit for the ratios of geometric means of values in 2 conditions.	Unpaired and paired samples. Number of variables: use it if there are thousands or hundreds of isotopologues. Do not use it for fractional contributions or total metabolite abundances if you have few (< 100) metabolites.
	prm-scipy	Permutations method via scipy	Two populations are drawn from the same underlying distribution.	Differences between means of subsampled values from 2 conditions.	Unpaired and paired samples. Any number of variables.
multi-group	KW	Kruskal-Wallis	Three or more populations share the same medians.	Based on the difference in the group-wise order totals.	Independent samples; a.k.a "the non-parametric alternative to the ANOVA (analysis of variance) test". Works better with $n \geq 5$. Any number of variables.

Supplementary Table 1: Non-parametric statistical tests offered by type of univariate analyses. All these tests are rank-based with the exception of disfit and prm-scipy. Visit https://docs.scipy.org/doc/scipy/reference/generated/scipy.stats.permutation_test.html for more information about scipy permutations function.

Spearman or the Pearson correlation test. The Spearman test computes the correlation coefficients (ρ) based on the sum of the squared differences between the paired ranks, and the p-values are computed via the t-statistic for each ρ value. In the Pearson test, the correlation coefficients (r) are computed by linear regression, and the p-values are estimated via the t statistic. As a rule of thumb, the Spearman test is recommended, and this is the option set by default in DIMet.

**Type of analysis**	**Null hypothesis**	**Principle**
MDV profile comparison between 2 conditions	The MDV profiles of the two conditions are not correlated	Using the set of values of the isotopologue proportions that correspond to the metabolite MDV, performs a linear regression between the set of values of the first condition and the set of values of the second condition.
MDV profile comparison between 2 consecutive time-points	The MDV profiles of the two time-points are not correlated	Same as above, but between the first and the second consecutive time-points.
Metabolite total abundances and fractional contribution time-course profile comparison between 2 conditions	The time-course profiles of the two conditions are not correlated	Using the metabolite total abundance or fractional contribution, performs a linear regression between the set of values (matched across the time-points) of the first condition and the second condition.

Supplementary Table 2: The offered bi-variate analyses. Note that the set of values in each case is obtained by computing the geometric means across the biological replicates. The Spearman correlation test is run for each type of bi-variate analysis.

S3.3 Multiple tests correction methods

The correction for multiple tests is available for both univariate and bi-variate analyses using either Bonferroni ("bonferroni") or Benjamini-Hochberg ("fdr_bh") correction. A general rule of thumb to use between the two methods is the following. If a stringent method is preferred and the inflation of false negatives is not a concern, Bonferroni method is recommended. In contrast, if the priority is to reduce the frequency of false negatives, the Benjamini-Hochberg method is recommended. The option set by default in DIMet is Benjamini-Hochberg ("fdr_bh").
For more information, see statsmodels.

S3.4 Output table of the DIMet univariate analyses

For each analysis DIMet generates tabular delimited files as output. Supplementary table 3 provides details on the content of the columns.

Note: The distance/span (d/s) is the measure of the distance between two intervals of values (corresponding to the compared groups), normalized by the global span of all the values. This metric takes values between -1 to 1. Negative d/s values indicate an overlap, which means that this variable (metabolite) can not be a biomarker to distinguish between groups. Positive d/s values show the distance between intervals, the closer to 1, the greater. Groups that do not overlap reflect a reproducible difference and an indication that this variable is a potential biomarker. This must be interpreted alongside the adjusted p-values and the Fold Changes. The d/s is sensitive to outliers, if these are present, careful interpretation is required.

S3.5 Output table of the DIMet bi-variate analyses

The output table of the bi-variate analysis, performed with the chosen correlation test (Spearman or Pearson), contains the columns that are described in the Supplementary table 4.

Column name	Description/Interpretation
log2FC	log-transformed fold changes base 2.
pvalue	computed by the chosen statistical test
padj	adjusted p-values obtained by the chosen multiple correction method.
distance/span	the normalized distance between the intervals of the two groups (see text)
FC	fold changes between geometric means
count_nan_samples_group1	number of values missing in group 1 of samples.
count_nan_samples_group2	number of values missing in group 2 of samples.
distance	numerator in the distance/span ratio
span_allsamples	denominator in the distance/span ratio
compartment	extracellular, cellular, or specific subcellular compartment
gmean_1	geometric mean of values from samples in group 1
gmean_2	geometric mean of the values from samples in group 2

Supplementary Table 3: Columns of the output file for univariate analyses

Column name	Description/Interpretation
correlation_coefficient	The correlation_coefficient is a value between -1 and 1
pvalue	Computed by the correlation test
padj	The adjusted p-values obtained by the chosen multiple correction method
gmean_arr_1	For each metabolite, the array of the geometric means that correspond to the first group
gmean_arr_2	For each metabolite, the array of the geometric means that correspond to the second group

Supplementary Table 4: Columns of the output file for bi-variate analyses.
